# Social Capital and COVID-19 Deaths: An Ecological Analysis in Japan

**DOI:** 10.3390/ijerph182010982

**Published:** 2021-10-19

**Authors:** Hiroshi Murayama, Isuzu Nakamoto, Takahiro Tabuchi

**Affiliations:** 1 Research Team for Social Participation and Community Health, Tokyo Metropolitan Institute of Gerontology, Tokyo 173-0015, Japan; nakamoto@tmig.or.jp; 2 Cancer Control Center, Osaka International Cancer Institute, Osaka 541-8567, Japan; tabuti-ta@mc.pref.osaka.jp

**Keywords:** COVID-19, deaths, social capital, ecological analysis, Japan

## Abstract

Social contextual factors could determine mortality by the coronavirus disease 2019 (COVID-19), with social capital as a potential determinant. This study aimed to examine the association between prefecture-level social capital and COVID-19 deaths in Japan. Data on the cumulative number of COVID-19 deaths per 100,000 individuals between 1 October 2020 and 30 June 2021 in 47 prefectures were obtained from the government open-access database. Prefecture-level social capital was collected from a large-scale web-based nationwide survey conducted between August and September 2020. We included trust in neighbors, norm of reciprocity in the neighborhood, and trust in the national government as cognitive social capital, and neighborhood ties and social participation as structural social capital. The cumulative COVID-19 deaths per 100,000 individuals (1 October 2020 to 30 June 2021) ranged from 0.15 to 27.98 in 47 prefectures. A multiple regression analysis after adjusting for covariates showed that a greater norm of reciprocity and government trust were associated with fewer COVID-19 deaths during the first and second 3-month periods of observation. In the third 3-month period, the association between COVID-19 deaths and government trust became nonsignificant. Trust in neighbors, neighborhood ties, and social participation were not related to COVID-19 deaths during any time period. The disparity of COVID-19 deaths by prefecture in Japan can be explained by cognitive social capital. This study suggests that the association between social capital and COVID-19 deaths may vary according to the dimension of social capital and time period.

## 1. Introduction

The coronavirus disease 2019 (COVID-19) has spread worldwide since the first case was reported in Wuhan, China, in 2019. More than 4.5 million deaths due to COVID-19 were reported by 31 August 2021, worldwide [1]. Several factors related to COVID-19 deaths have been reported. Du et al. [2] conducted a meta-analysis and found that older age, male sex, chronic respiratory diseases, diabetes, hypertension, chronic kidney disease, and cardiovascular diseases were risk factors for death by COVID-19. In addition, Latino/African American people [3], smokers [4,5], obese people [6], and frail people [7] were also more likely to die due to COVID-19. In addition to these individual-level factors, some studies have demonstrated variations in the number of COVID-19 deaths by country or community [8,9]. This implies that social contextual factors could determine COVID-19 deaths, with social capital as a potential determinant.

Social capital has been examined in various academic fields. Public health researchers, particularly those in the field of social epidemiology, have explored the association between social capital and health. According to Putnam [10], the term refers to “features of social organization, such as trust, norms, and networks that can improve the efficacy of society by facilitating coordinated actions”. Kawachi and Berkman [11] defined social capital as resources that are available to individuals as a result of their membership in a group/community.

In a time of crisis, higher social capital in the community enhances individuals’ and communities’ abilities of preparation, response, and recovery toward crisis by enabling easier access to various resources, such as information, help, and physical/financial resources [12]. To date, there is some evidence that community social capital is associated with COVID-19 deaths [13,14,15,16,17,18,19] (Table 1). For example, Makridis and Wu [13] used data for counties in the United States (U.S.) and reported that county-level social capital, which contains comprehensive indicators, such as family stability and interaction, social trust, confidence in institutions, community cohesion, and volunteerism were associated with lower COVID-19 deaths.

However, the association between community social capital and COVID-19 deaths remains controversial in at least two aspects. First, the association (e.g., direction and magnitude) varied across the dimensions of social capital. Yanagisawa et al. [14] revealed that U.S. counties with higher social/emotional support experienced fewer COVID-19 deaths, while counties with higher civic participation had more COVID-19 deaths. Imbulana Arachchi and Managi [15] used data from 37 countries and reported that community attachment and social trust were associated with more COVID-19 deaths, while family bonds and neighborhood security were associated with fewer deaths. Second, the association changed over time. As the COVID-19 pandemic is an unprecedented crisis, it is difficult to select measures to control COVID-19. Moreover, people’s behaviors and perceptions towards COVID-19 tend to be erratic. Borgonovi et al. [16] showed that the association between social capital and COVID-19 outcomes, including death cases, varied considerably over time. Other studies have reported the same trend [17,18]. Therefore, studies should consider the dimensions of social capital and the time period in qualifying the association.

In addition, the influence of social capital on health depends on contexts, such as socioeconomic level [20] and inequality level [21]. Previous studies regarding the association between social capital and COVID-19 deaths were derived from Western countries or used world data. However, the findings from these studies may be different from those in non-Western countries, such as Japan. In Japan, a relatively collectivist society with strong group ties, residents within a community feel comfortable under systems of mutual assurance and monitoring [22,23,24]. Considering the differences in background between the populations of Western countries and Japan, it is important to examine the influence of social capital on COVID-19 deaths in Japan.

Given this background, this study aimed to examine the association between prefecture-level social capital and COVID-19 deaths in Japan. We hypothesized that prefectures with higher social capital would exhibit a lower number of COVID-19 deaths.

## 2. Materials and Methods

### 2.1. Study Design and Data Sources

This was an ecological study based on data derived from several open-access databases and a survey conducted in Japan. We collected the number of COVID-19 deaths in Japan from the website, “Visualizing the Data: Information on COVID-19 Infections” [25]. This is an open-access database managed by the Ministry of Health, Labour, and Welfare. The first death in Japan occurred on 13 February 2020. We identified 16,080 COVID-19 deaths in Japan as of 31 August 2021 [1]. Figure S1 presents the number of COVID-19 death cases reported daily in Japan until 31 August 2021 [1].

The Japanese government has declared a state of emergency (mild lockdown measures). In Tokyo, until September 2021, a state of emergency has been declared four times: (i) from 7 April to 25 May 2020, (ii) from 8 January to 21 March 2021, (iii) from 25 April to 20 June 2021, and (iv) from 12 July to 30 September 2021. In addition, although the government has not forced social distancing on people, they conducted a nationwide campaign regarding social distancing, named “Avoid the Three Cs” (closed spaces, crowded places, and close-contact settings).

Social capital data were collected from the Japan “COVID-19 and Society” Internet Survey (JACSIS) study, conducted in 2020. The JACSIS study was a national-representative, web-based, self-reported questionnaire survey that employed a large internet survey agency (Rakuten Insight, Inc., Tokyo, Japan). A total of 28,000 people aged 15–79 years were included in the survey. The questionnaire was distributed between 25 August and 30 September 2020. The survey period was during the latter half of the second wave of the pandemic in Japan (between July and September 2020). After validating the data quality, we excluded 2518 respondents with discrepant or artificial/fraudulent responses and included 25,482 respondents in the analysis. This study was reviewed and approved by the Research Ethics Committee of the Osaka International Cancer Institute (approved on 19 June 2020; approval number 20084).

Information on covariates was collected from the Population Estimates (as of October 2019) [26] by the Statistics Bureau of Japan, and the Basic Survey on Wage Structure in 2019 [27] and the Hospital Report (as of October 2019) [28] by the Ministry of Health, Labour, and Welfare, respectively.

### 2.2. Measures

#### 2.2.1. Death by COVID-19

We calculated the cumulative number of COVID-19 deaths per 100,000 individuals in 47 prefectures from 1 October to 31 December 2020 (first 3-month period from the JACSIS survey), from 1 January to 31 March 2021 (second 3-month period from the survey), and from 1 April to 30 June 2021 (third 3-month period from the survey).

#### 2.2.2. Social Capital

We used five social capital indicators in this study which included the following: (i) trust in neighbors, (ii) norm of reciprocity in the neighborhood, (iii) trust in the national government, (iv) neighborhood ties, and (v) social participation. Neighborhood trust, norm of reciprocity, and trust in the government were measured as the cognitive dimension of social capital, and neighborhood ties and social participation as structural dimensions.

Trust in neighbors, norm of reciprocity in the neighborhood, and trust in the national government were assessed with one item, respectively, with the statements of “People in your neighborhood can be trusted”, “People in your neighborhood help each other”, and “National government can be trusted”. Possible answers were: “1 = agree”, “2 = somewhat agree”, “3 = somewhat disagree”, or “4 = disagree”. We classified the responses into two categories: agree (responses of 1 and 2) and disagree (responses of 3 and 4). Neighborhood ties referred to the frequency of connection with neighbors. Respondents answered this item using a seven-point scale: “1 = never”, “2 = once a month”, “3 = 2–3 times in a month”, “4 = once a week”, “5 = 2–3 times in a week”, “6 = 4–5 times in a week”, or “7 = almost every day (6–7 times in a week)”. Responses were dichotomized into less than once per week (sparse; responses of 1–3) and more than once in a week (dense; responses of 4–7). Finally, concerning social participation, we asked respondents whether they participated in activities related to the following three groups: volunteering, sports, and hobbies. If they selected at least one group, we considered them as participating in the activity.

The responses for each social capital item were aggregated within the residential prefectures. We calculated the proportions of people who agreed to the items of neighborhood trust, norm of reciprocity in the neighborhood, and trust in the national government, who had dense neighborhood ties, and who participated in any activity, by 47 prefectures, using inverse probability weighting. Weights were calculated by logistic regression analysis using sex, age, and socioeconomic factors to adjust for differences between the respondents of the present internet survey and the respondents in a widely used population-based sample that is representative of the Japanese population from the 2016 Comprehensive Survey of Living Conditions [29].

#### 2.2.3. Covariates

We used the following four indexes: proportion of people aged ≥65 years and population density from the Population Estimates [26], average monthly income level from the Basic Survey on Wage Structure in 2019 [27], and the number of hospital beds per 100,000 individuals from the Hospital Report [28]. This information was calculated by prefectures.

### 2.3. Statistical Analyses

A multiple regression analysis was performed, with the cumulative number of COVID-19 deaths as a dependent variable. Before performing the analysis, we calculated correlation coefficients (Pearson’s r) among the variables of social capital and covariates to check for multicollinearity. Because the variance in each variable could potentially be large due to the small sample size, we divided all the variables (i.e., social capital and covariates) into tertiles and used them in the regression analysis. The five social capital indicators were separately added into the model as independent variables with adjustment for covariates. The results are expressed as regression coefficients (b) with 95% confidence intervals (CIs). The analysis was performed using IBM SPSS 23 (IBM Corp., Armonk, NY, USA).

## 3. Results

**Figure 1:**
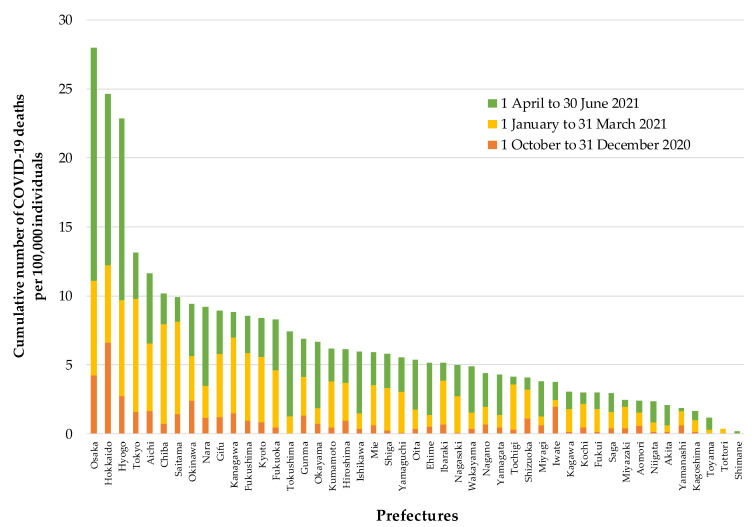
Distribution of the cumulative number of COVID-19 deaths per 100,000 individuals from 1 October 2020 to 30 June 2021, in 47 prefectures in Japan.

Figure 1 shows the distribution of the cumulative number of COVID-19 deaths per 100,000 individuals from 1 October 2020 to 30 June 2021, in 47 prefectures. The highest was 27.98 and the lowest was 0.15. Table S1 shows the cumulative number of COVID-19 deaths per 100,000 individuals for each 3-month period (1 October to 31 December 2020, 1 January to 31 March 2021, and 1 April to 30 June 2021) as well as during the total observational period (1 October 2020 to 30 June 2021).

Table 2 presents the descriptive statistics of the social capital indicators, covariates, and their correlations. The correlation coefficients among the social capital indicators ranged from 0.035 to 0.759. As the proportion of people aged ≥65 years was strongly correlated with population density and the number of hospital beds (r = −0.609 and 0.633), we did not use the proportion of people aged ≥65 years as a covariate in the regression model to avoid multicollinearity.

Table 3 shows the results of multiple regression analysis for the three time periods after adjusting for population density, average monthly income level, and the number of hospital beds. Greater norms of reciprocity and trust for the national government were associated with fewer COVID-19 deaths during the first and second 3 months (1 October to 31 December 2020 and 1 January to 31 March 2021). In the third 3-month period (1 April to 30 June 2021), the association of the norm of reciprocity with COVID-19 death was unchanged, but that of government trust was attenuated and became nonsignificant. Trust in neighbors, neighborhood ties, and social participation were not significantly related to COVID-19 deaths during any time period.

## 4. Discussion

This study examined the association between prefecture-level social capital and COVID-19 deaths in Japan. We identified that the norm of reciprocity was associated with fewer COVID-19 deaths throughout the observation period. Trust in the national government was linked to fewer COVID-19 deaths in the first and second 3-month periods, but not in the third 3-month period. Neighborhood trust, neighborhood ties, and social participation were not consistently associated with COVID-19 deaths. As previous studies were based on data from Western countries or world data, this is the first finding specific to Asian nations regarding the relationship between social capital and COVID-19 deaths. This study confirmed that the association of social capital with COVID-19 deaths varied by the dimension of the social capital indicator and time period, as indicated by previous studies [16,17,18].

The norm of reciprocity was consistently associated with COVID-19 deaths in the short-, medium-, and long-term assessments. Yanagisawa et al. [14] reported that higher social and emotional support in the community was correlated with fewer COVID-19 deaths. People developed greater anxiety and depression during the COVID-19 pandemic [30], and the pandemic has increased mental health disparities [31]. A mutual helping system in the community may be able to relieve people’s stress, and thus enable people to adapt to the isolation of stay-at-home orders. Another plausible explanation for the association is mutual monitoring (or informal guardianship) in the community. Deviant behaviors related to COVID-19 (e.g., no mask-wearing, no vaccination) could be inhibited through informal social control. As those living in such communities tend to work harder to maintain social order, they might step in to intervene in the community when they witness others engaging in deviant behaviors [11].

Trust in the government was associated with COVID-19 deaths during the earlier period of observation, but not in the later period. A previous study reported that lower government trust was associated with lower compliance with infection control policies and less practice of preventive behaviors during the Ebola crisis [32,33], as well as during the recent COVID-19 pandemic [34]. Individuals living in communities with a higher level of government trust may be more adherent to COVID-19-related policies, such as lockdown and social distancing. However, trust in the government was also time-variant. Indeed, during the observational period, the current Japanese Cabinet approval rating had been decreasing: the approval rating was higher than the disapproval rating until December 2020, but disapproval exceeded approval in 2021 [35]. This finding indicates that although government trust could affect the rate of COVID-19 deaths, it did not necessarily predict long-term outcomes.

Higher trust in neighbors was not associated with COVID-19 death. The association between social trust and COVID-19 deaths seems to be complicated. From the perspective of a widely accepted hypothesis that social capital affects health, residing in a community with high trust can be associated with people’s better health status through psychological relief [11]. On the contrary, it has been reported that societies with high trust might be more vulnerable to deception about the severity of COVID-19, counterfeit treatments, and contemptuous perspectives on physical distancing [36]. Previous studies have shown a relationship between higher community social trust and higher COVID-19 mortality [15,17]. There is a need to accumulate more findings to understand this association in detail.

Regarding structural social capital, neither indicators (i.e., neighborhood ties and social participation) were associated with COVID-19 deaths. A previous article reported that greater civic engagement was positively related to COVID-19 mortality [14]. This was because, in the community with greater civic engagement, residents tended to have frequent communal in-person gatherings. On the contrary, structural social capital can contribute to better health. For instance, people living in such areas can easily and quickly obtain information and knowledge about infection prevention in terms of COVID-19 through their tightly knit neighborhood networks and their frequent opportunities for community activities (called “social contagion”) [11]. These possible positive and negative effects of social capital on COVID-19 may offset each other and thus, no association was observed in this study.

We measured social capital based on a survey conducted during the COVID-19 pandemic. In contrast, all previous studies on the relationship between social capital and COVID-19 deaths used social capital indicators measured before the pandemic [13,14,15,16,17,18,19]. Social capital may change over time [37]. Policies related to COVID-19 prevention, such as social distancing and lockdown would presumably influence social capital. Therefore, to develop strategies to foster community social capital in the COVID-19 era, it is also important to focus on social capital during the pandemic.

This study has some limitations. First, there were several potentially uncontrolled confounding factors. We could not fully adjust for the variables because of the small sample size. This was because the Ministry of Health, Labour, and Welfare released information on the number of COVID-19 deaths by prefecture, not the local municipality. Therefore, an analytic unit must be at the prefecture-level. Second, although we applied an ecological analysis, a multilevel analysis would be a better approach to avoid ecological fallacy. However, at this time in Japan, it is difficult to obtain information on individual COVID-19 deaths from government open-access data. Future analyses should consider utilizing data regarding the individual causes of death.

## 5. Conclusions

This study suggests that prefectures with greater cognitive social capital—the norm of reciprocity and trust for the national government—experienced fewer COVID-19 deaths in Japan. The norm of reciprocity was associated with COVID-19 deaths throughout the observational period, and government trust was only associated with COVID-19 deaths in the short and medium terms. Social capital, particularly cognitive social capital, could play an important role during the COVID-19 pandemic in Japanese communities, and the disparity of COVID-19 deaths by the prefectures in Japan can be explained by social capital. The findings also suggest that the association between social capital and COVID-19 deaths may vary according to the dimension of social capital and time period.

## Figures and Tables

**Table 1:** Previous findings on the relationship between social capital and COVID-19 deaths.

Author, Year	Data (Analytic Sample)	Outcome	Social Capital Variable	Findings
Borgonovi et al., 2021 [16]	United States (2284 counties)	Death cases	Relational social capital	↓
			Cognitive social capital	↓ (early period)/↑ (middle/late period)
Fraser et al., 2021 [18]	United States (947 counties)	Excess death cases	Bonding social capital	↓ (early period)
			Bridging social capital	↓ (middle period)/↑ (late period)
			Linking social capital	↓ (middle period)
Makridis & Wu, 2021 [13]	United States (over 2700 counties)	Death cases	Aggregated indicator (including social trust, community cohesion, volunteerism, facility interaction and investment, etc.)	↓
Yanagisawa et al., 2021 [14]	United States (nationwide counties; no description on the number)	Death cases	Social/emotional support	↓
			Engagement in voluntary organizations	No association
			Civic engagement	↑ (only on voter turnout)
Bartscher et al., 2020 [19]	European nations (over 800 areas from four countries)	Excess death cases	Voter turnout	↓
Elgar et al., 2021 [17]	Worldwide (84 countries)	Death cases	Social trust	↑
			Group affiliation	↑
			Civic engagement	↓
			Confidence in institution	↓
Imbulana Arachchi & Managi, 2021 [15]	Worldwide (29 countries and 265 province/state from eight countries)	Death cases	Community attachment	↑
			Social trust	↑
			Family bond	↓
			Neighborhood security	↓

↑: increase COVID-19 deaths. ↓: decrease COVID-19 deaths.

**Table 2:** Descriptive statistics of social capital and covariates and their correlations.

Variable	Mean ± SD (Min–Max; Median)	Pearson’s r								
		a	b	c	d	e	f	g	h	i
a. Trust in neighbors (%)	65.9 ± 2.9 (61.1–72.5; 65.4)		0.759 *	0.349 *	0.365 *	0.235	0.353 *	−0.349 *	−0.042	0.210
b. Norm of reciprocity in the neighborhood (%)	56.2 ± 3.2 (51.1–63.7; 55.5)			0.457 *	0.403 *	0.035	0.394 *	−0.397^*^	−0.181	0.226
c. Trust in the national government (%)	44.3 ± 3.3 (37.4–53.7; 44.2)				0.297 *	0.091	0.348 *	−0.227	−0.175	0.287
d. Neighborhood ties (%)	12.3 ± 2.7 (7.3–18.6; 12.3)					0.542 *	0.440 *	−0.422 *	−0.211	0.181
e. Social participation (%)	23.9 ± 2.7 (17.9–29.5; 24.0)						0.115	−0.078	−0.160	0.088
f. Proportion of people aged ≥65 years (%)	30.5 ± 3.1 (22.2–37.3; 30.8)							−0.609 *	−0.184	0.633 *
g. Population density (persons/km^2^)	656.2 ± 1216.5 (66.9–6354.8; 265.9)								0.395 *	−0.417 *
h. Average monthly income (thousand yen)	280.5 ± 27.8 (239–379; 280.6)									−0.135
i. Number of hospital beds (per 100,000 individuals)	1398.6 ± 360.8 (805–2508; 1366)									

SD: standard deviation; *: statistically significant (*p* < 0.05).

**Table 3:** Association between social capital and COVID-19 deaths by time period.

Variable	Category	COVID-19 Deaths from 1 October to 31 December 2020	COVID-19 Deaths from 1 January to 31 March 2021	COVID-19 Deaths from 1 April to 30 June 2021
		b (95% CI)	b (95% CI)	b (95% CI)
Trust in neighbors	1st tertile (low)	Reference	Reference	Reference
	2nd tertile (middle)	0.09 (−0.79, 0.98)	0.28 (−1.06, 1.62)	0.93 (−1.55, 3.40)
	3rd tertile (high)	−0.65 (−1.54, 0.25)	−0.66 (−2.00, 0.69)	−1.15 (−3.64, 1.34)
Norm of reciprocity	1st tertile (low)	Reference	Reference	Reference
	2nd tertile (middle)	−0.89 (−17.60, −0.19)	−0.38 (−1.70, 0.94)	−1.64 (−4.13, 0.86)
	3rd tertile (high)	−1.10 (−2.03, −0.17)	−1.43 (−2.84, −0.03)	−2.54 (−5.20, −0.02)
Trust in the national government	1st tertile (low)	Reference	Reference	Reference
	2nd tertile (middle)	−0.29 (−1.16, 0.58)	−1.24 (−2.53, −0.05)	1.09 (−1.38, 3.57)
	3rd tertile (high)	−0.84 (−1.73, −0.04)	−1.20 (−2.51, −0.01)	−0.73 (−3.24, 1.79)
Neighborhood ties	1st tertile (low)	Reference	Reference	Reference
	2nd tertile (middle)	−0.28 (−1.21, 0.64)	−0.56 (−1.87, 0.75)	1.74 (−0.83, 4.31)
	3rd tertile (high)	−0.81 (−1.71, 0.10)	−1.23 (−3.01, 0.45)	−0.33 (−2.85, 2.19)
Social participation	1st tertile (low)	Reference	Reference	Reference
	2nd tertile (middle)	−0.72 (−1.60, 0.17)	−0.83 (−2.15, 0.48)	−0.22 (−2.77, 2.33)
	3rd tertile (high)	−0.53 (−1.43, 0.38)	−1.18 (−2.53, 0.16)	−0.57 (−3.18, 2.03)

CI: confidence interval. Adjusted for the proportion of population density, average monthly income level, and the number of hospital beds per 100,000 individuals. Each social capital indicator was separately added to the model.

## Data Availability

The datasets used and analyzed in the current study are available from the corresponding author upon reasonable request.

## Supplementary Materials

The following are available online at https://www.mdpi.com/article/10.3390/ijerph182010982/s1, Figure S1: The transition of the number of COVID-19 deaths through 31 August 2021, in Japan; Table S1: Cumulative number of COVID-19 deaths by prefectures in Japan.
