# Supplementary material for: Social Capital and COVID-19 Deaths: An Ecological Analysis in Japan
Source: Int J Environ Res Public Health. 2021 Oct 19;18(20):10982. doi: 10.3390/ijerph182010982 (PMC8536097; doi:10.3390/ijerph182010982)
Supplement: Supplementary file 1 [file ijerph-18-10982-s001.zip › ijerph-1400067-supplementary.pdf]

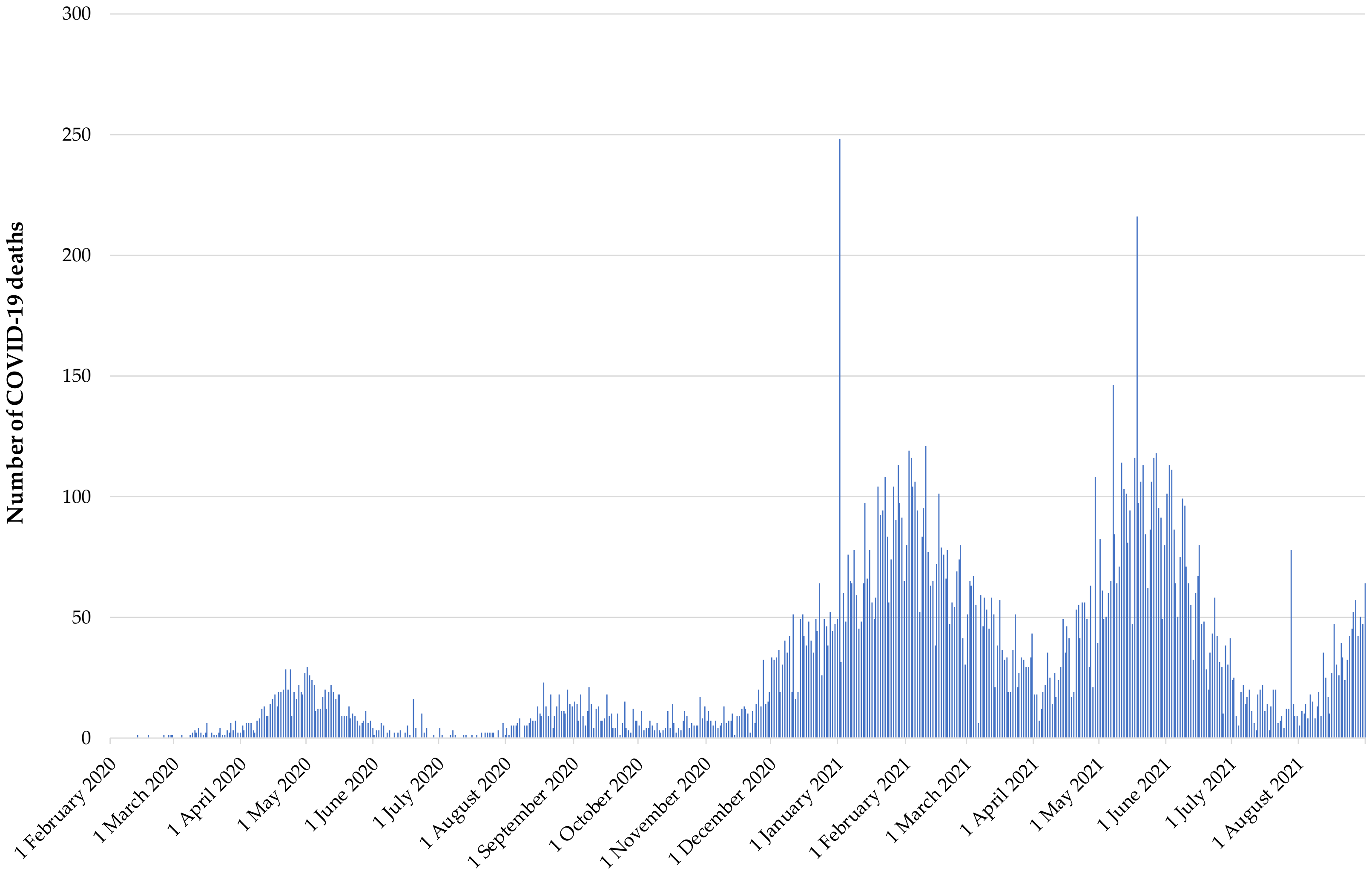

Figure S1. The transition of the number of COVID-19 deaths through 31 August 2021 in Japan

Table S1. Cumulative number of COVID-19 deaths by prefectures in Japan

Prefecture	Population (thousand persons)	COVID-19 deaths per 100,000 individuals			
		1 October to 31 December 2020	1 January to 31 March 2021	1 April to 30 June 2021	1 October 2020 to 30 June 2021 (Total period)
Total	126,167	1.50	4.52	4.45	10.47
Hokkaido	5,250	6.59	5.64	12.38	24.61
Aomori	1,246	0.56	0.96	0.88	2.41
Iwate	1,227	1.96	0.49	1.30	3.75
Miyagi	2,306	0.61	0.65	2.56	3.82
Akita	966	0.10	0.52	1.45	2.07
Yamagata	1,078	0.46	0.93	2.88	4.27
Fukushima	1,846	0.92	4.93	2.71	8.56
Ibaraki	2,860	0.66	3.18	1.29	5.14
Tochigi	1,934	0.26	3.31	0.57	4.14
Gunma	1,942	1.29	2.83	2.73	6.85
Saitama	7,350	1.41	6.72	1.78	9.92
Chiba	6,259	0.73	7.21	2.20	10.15
Tokyo	13,921	1.57	8.21	3.33	13.12
Kanagawa	9,198	1.47	5.52	1.82	8.81
Niigata	2,223	0.13	0.67	1.53	2.34
Toyama	1,044	0.00	0.29	0.86	1.15
Ishikawa	1,138	0.35	1.14	4.48	5.98
Fukui	768	0.13	1.69	1.17	2.99
Yamanashi	811	0.62	0.99	0.25	1.85
Nagano	2,049	0.68	1.27	2.44	4.39
Gifu	1,987	1.21	4.58	3.12	8.91
Shizuoka	3,644	1.07	2.11	0.88	4.06
Aichi	7,552	1.66	4.91	5.05	11.61
Mie	1,781	0.62	2.92	2.36	5.90
Shiga	1,414	0.21	3.11	2.48	5.80
Kyoto	2,583	0.85	4.72	2.83	8.40
Osaka	8,809	4.23	6.87	16.88	27.98
Hyogo	5,466	2.74	6.92	13.17	22.83

Nara	1,330	1.13	2.33	5.71	9.17
Wakayama	925	0.32	1.19	3.35	4.86
Tottori	556	0.00	0.36	0.00	0.36
Shimane	674	0.00	0.00	0.15	0.15
Okayama	1,890	0.69	1.16	4.81	6.67
Hiroshima	2,804	0.93	2.75	2.46	6.13
Yamaguchi	1,358	0.07	2.95	2.50	5.52
Tokushima	728	0.00	1.24	6.18	7.42
Kagawa	956	0.10	1.67	1.26	3.03
Ehime	1,339	0.52	0.82	3.81	5.15
Kochi	698	0.43	1.72	0.86	3.01
Fukuoka	5,104	0.43	4.17	3.68	8.29
Saga	815	0.37	1.23	1.35	2.94
Nagasaki	1,327	0.08	2.64	2.26	4.97
Kumamoto	1,748	0.46	3.32	2.40	6.18
Oita	1,135	0.35	1.41	3.61	5.37
Miyazaki	1,073	0.37	1.58	0.47	2.42
Kagoshima	1,602	0.12	0.87	0.62	1.62
Okinawa	1,453	2.41	3.23	3.79	9.43
